# Using ^31^P-MRI of hydroxyapatite for bone attenuation correction in PET-MRI: proof of concept in the rodent brain

**DOI:** 10.1186/s40658-017-0183-6

**Published:** 2017-05-02

**Authors:** Vincent Lebon, Sébastien Jan, Yoann Fontyn, Brice Tiret, Géraldine Pottier, Emilie Jaumain, Julien Valette

**Affiliations:** 1 Commissariat à l’Energie Atomique et aux Energies Alternatives (CEA), Direction de la Recherche Fondamentale (DRF), Institut d’Imagerie Biomedicale (I2BM), MIRCen, Fontenay-aux-Roses, France; 2 Centre National de la Recherche Scientifique (CNRS), Neurodegenerative Diseases Laboratory, Université Paris-Sud, Université Paris-Saclay, UMR 9199 Fontenay-aux-Roses, France; 3 Commissariat à l’Energie Atomique et aux Energies Alternatives (CEA), Direction de la Recherche Fondamentale (DRF), Institut d’Imagerie Biomedicale (I2BM), SHFJ, Orsay, France; 4 Inserm/CEA/Université Paris Sud, Université Paris-Saclay, UMR 1023—CNRS ERL 9218, IMIV, Orsay, France

**Keywords:** PET/MR, Attenuation correction, ^31^P, ZTE, Bone

## Abstract

**Background:**

The correction of γ-photon attenuation in PET-MRI remains a critical issue, especially for bone attenuation. This problem is of great importance for brain studies due to the density of the skull. Current techniques for skull attenuation correction (AC) provide indirect estimates of cortical bone density, leading to inaccurate estimates of brain activity. The purpose of this study was to develop an alternate method for bone attenuation correction based on NMR.

The proposed approach relies on the detection of hydroxyapatite crystals by zero echo time (ZTE) MRI of ^31^P, providing individual and quantitative assessment of bone density. This work presents a proof of concept of this approach. The first step of the method is a calibration experiment to determine the conversion relationship between the ^31^P signal and the linear attenuation coefficient μ. Then ^31^P-ZTE was performed in vivo in rodent to estimate the μ-map of the skull. ^18^F-FDG PET data were acquired in the same animal and reconstructed with three different AC methods: ^31^P-based AC, AC neglecting the bone and the gold standard, CT-based AC, used to comparison for the other two methods.

**Results:**

The calibration experiment provided a conversion factor of ^31^P signal into μ. In vivo ^31^P-ZTE made it possible to acquire 3D images of the rat skull. Brain PET images showed underestimation of ^18^F activity in peripheral regions close to the skull when AC neglected the bone (as compared with CT-based AC). The use of ^31^P-derived μ-map for AC leads to increased peripheral activity, and therefore a global overestimation of brain ^18^F activity.

**Conclusions:**

In vivo ^31^P-ZTE MRI of hydroxyapatite provides μ-map of the skull, which can be used for attenuation correction of ^18^F-FDG PET images. This study is limited by several intrinsic biases associated with the size of the rat brain, which are unlikely to affect human data on a clinical PET-MRI system.

## Background

Five years after the introduction of the first whole-body PET/MR systems, attenuation correction of PET images remains challenging. This is particularly true for bone attenuation, due to the inability of ^1^H-MRI to detect the crystal component of cortical bone. Neglecting bone attenuation leads to significant activity underestimation in bone as well as in neighboring tissues [1, 2], which is of particular concern for the brain given the skull density. Original approaches have been developed to assess skull attenuation by MRI, such as atlas-based, template-based, and segmentation-based methods [3]. However, these approaches provide an indirect assessment of bone density and remain inaccurate in the vicinity of bones [4, 5]. In this context, the ability to measure the density of cortical bone crystal in vivo by MRI would be of great interest. This study presents a new MR-based method for bone attenuation correction, based on ^31^P-MRI of hydroxyapatite crystals. The method was implemented on preclinical MRI and PET/CT systems in order to provide in vivo proof of concept in rodent. A calibration experiment was first conducted on a bone sample to determine the conversion factor from ^31^P signal intensity to linear attenuation coefficient μ. Then in vivo MRI and PET/CT were performed on a rat. A μ-map of the skull was calculated from ^31^P-MRI and used to correct ^18^F-FDG PET images of the brain for skull attenuation.

### Rationale

**Fig. 1:**
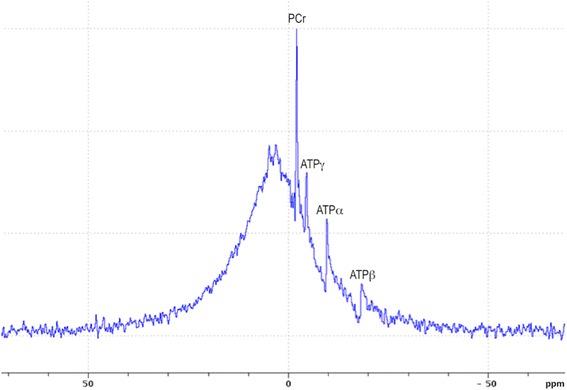
^31^P-NMR spectrum of a rat head in vivo

Bone crystal is made of hydroxyapatite Ca_5_(PO_4_)_3_OH [6], which high density (3.8 kg/L) and high atomic number (*Z* = 20 for Ca) explain γ-photon attenuation. Hydroxyapatite is hardly detectable by ^1^H-MRI in vivo. However, each crystal contains three phosphorus atoms that are detectable by ^31^P-NMR. The NMR spectrum in Fig. 1 displays the total ^31^P-NMR signal acquired from a rat head in vivo at 11.7 T (Fourier-transformed FID, 15 μs hard pulse for excitation, TR = 15 ms, 66 averages). This spectrum shows that ^31^P signal is dominated by a broad peak, which can unambiguously be ascribed to solid-state ^31^P given its short T2 of ~85 μs (FWHM ~18.5 ppm) and long T1 of ~20 s (fitted from spectra acquired for five different TR, data not shown). In contrast, the sharp peaks at −2.5, −5.0, −10.0, and −18.8 ppm belong to the liquid-state phosphorus atoms of PCr and ATP [7]. Integration of the spectrum shows that the liquid signal accounts for 7% of total ^31^P signal of the head (ratio of ATP + PCr + Pi peak areas divided by the total ^31^P resonance area). Thus, signal from hydroxyapatite dominates the ^31^P-NMR signal from the head in vivo. Solid-state ^31^P MRI techniques, which are appropriate for the detection of nuclei having short T2, have been proposed for MRI of cortical bone [8–15]. Based on these observations, we hypothesized that ^31^P-ZTE imaging of the head in vivo will provide 3D images of the skull, which can be converted into an attenuation map and used to correct PET data for γ-photon attenuation.

### Methods

### Calibration experiment on a cortical bone sample

**Fig. 2:**
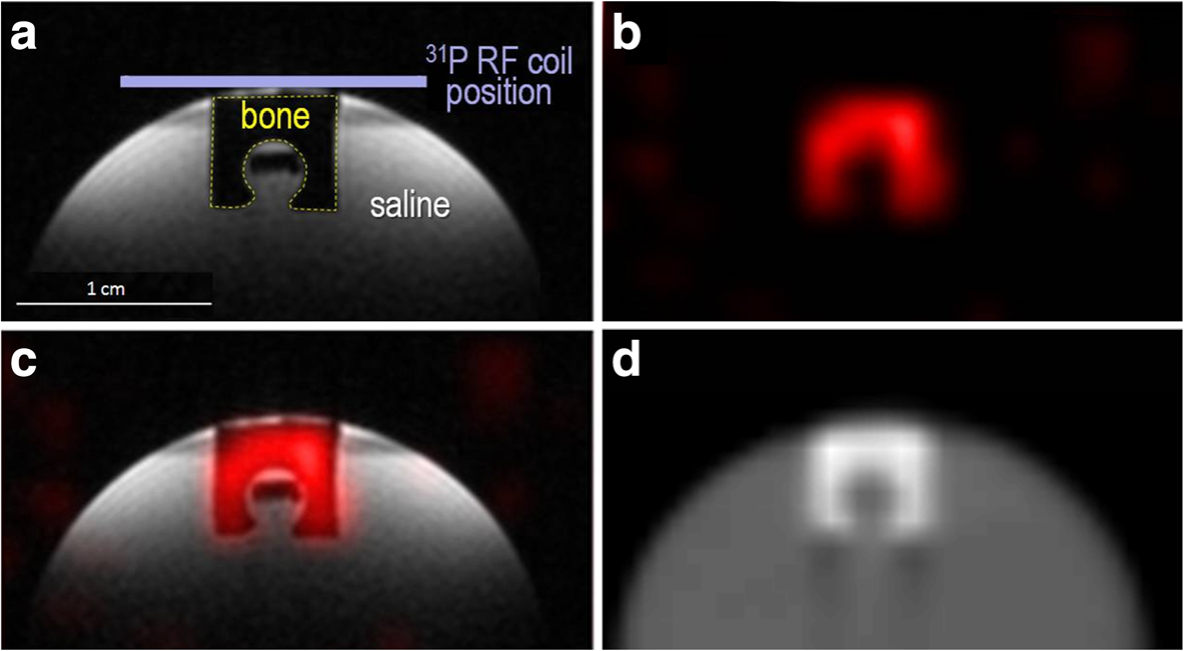
MRI and CT images acquired on the cortical bone sample. ^1^H anatomical MRI (**a**), ^31^P-ZTE MRI (**b**), fusion (**c**), and low-resolution CT (**d**). Note that the rectangular structure in hyposignal located in the center of the inverted U-shape bone in **a** is a piece of plastic used to position the bone sample on top of the tube

A U-shape sample was cut out of bovine cortical bone and positioned on top of a saline-filled tube, as shown in Fig. 2a. Saline was necessary for proper NMR shimming and frequency adjustment.

MRI data were acquired on a BRUKER BIOSPEC 117/16 USR system. Dedicated radiofrequency (RF) coils were placed on top of the sample (Fig. 2a), made of concentric ^1^H and ^31^P surface RF coils used for transmission and reception (15 mm diameter). After RF tuning/matching, shimming, and frequency adjustment, two images of the sample were acquired:

- ^1^H anatomical MRI (350 μm isotropic voxel size, proton density weighting) for registration of MRI and CT,
- ^31^P-ZTE MRI (1.4 mm isotropic voxel size), using the following parameters: 15 μs 200 W hard pulse for excitation, flip angle close to Ernst angle, 161mT/m readout gradient, 64 points sampled at 250 kHz, FOV = 89.6 mm, TR = 15 ms, 13030 half-projections, 96 averages, 5 h and 13 min acquisition time.

CT data were acquired on a SIEMENS INVEON system:

- High-resolution CT images of the rat head (103 μm isotropic) for registration of MRI and CT,
- Low-resolution CT images (860 × 860 × 796 μm) for μ-map generation.

^31^P-ZTE signal intensity was converted into ^31^P signal-to-noise ratio (SNR_31P_) by dividing the ^31^P signal intensity of each voxel by the standard deviation of the noise measured on a ROI distant from the bone sample. CT images were converted into μ-maps using the established bi-linear relationship between Hounsfield units and μ [16] and then registered to SNR_31P_ images. The conversion factor from SNR_31P_ to μ was obtained by calculating the mean μ/SNR_31P_ ratio. Note that the conversion factor is calculated for a given acquisition time of ^31^P-ZTE. Its use for ^31^P data acquired with different acquisition time will require correcting SNR_31P_ for the acquisition time (division by the square root of the acquisition time).

### Attenuation correction experiment on rodent head in vivo

A Sprague-Dawley male rat was used for this proof of concept and underwent MRI and PET/CT imaging sessions on the same systems as the bone sample. The PET/CT session was conducted on the rat 5 days after the MRI session. For the two sessions, the rat was positioned in a prone position and anesthetized by inhalation of a ~3% isoflurane/oxygen mixture. During the PET/CT session, the tail vein was catheterized for radiotracer injection. The protocol was approved by the Committee on the Ethics of Animal Experiments of the Commissariat à l’Energie Atomique.

The MRI session was identical to the one performed on the bone sample (acquisition of ^1^H anatomical MRI and ^31^P-ZTE MRI with identical parameters, except for ^31^P-ZTE acquisition which was limited to 16 averages, i.e., 52 min). During the PET/CT session, CT images were acquired with identical parameters as for the bone sample (high resolution for registration, low resolution for attenuation correction) and PET data were acquired in list mode for 60 min following an i.v. bolus of 93 MBq ^18^F-FDG.

^31^P-ZTE signal intensity was converted into SNR_31P_ and then into bone μ factor (“μ_31P_”) using the conversion factor determined on the bone sample (after correcting SNR for the different acquisition time). The PET and CT images were then registered to the μ_31P_ image.

A 1st set of PET images was reconstructed with attenuation correction based on CT generated μ-map (*CT-AC PET*): PET data were normalized, corrected for scattering, attenuation, arc effects, and physical decay, and reconstructed using a Fourier rebinning algorithm and an iterative OSEM-2D method.

A 2nd set of PET images with attenuation correction neglecting the bone (*NoBone-AC PET*) was obtained by assuming that the rat head was made of soft tissue and air only. NoBone-AC PET images provide a good approximation of Dixon-based attenuation correction.

A 3rd set of PET images was obtained by adding μ_31P_ to the attenuation map used for the 2nd set of PET images (^*31*^ *P-AC PET*). ^31^P-AC PET images illustrate the interest of ^31^P-ZTE over conventional Dixon-based approach.

## Results

Figure 2 presents the MRI and CT data acquired on the bone sample, demonstrating excellent co-localization between cortical bone and ^31^P-ZTE signal, as well as high SNR_31P_ of cortical bone (~30). The conversion factor measured on the sample was μ/SNR_31P_ = 0.0042 ± 0.0004 (cm^−1^).

**Fig. 3:**
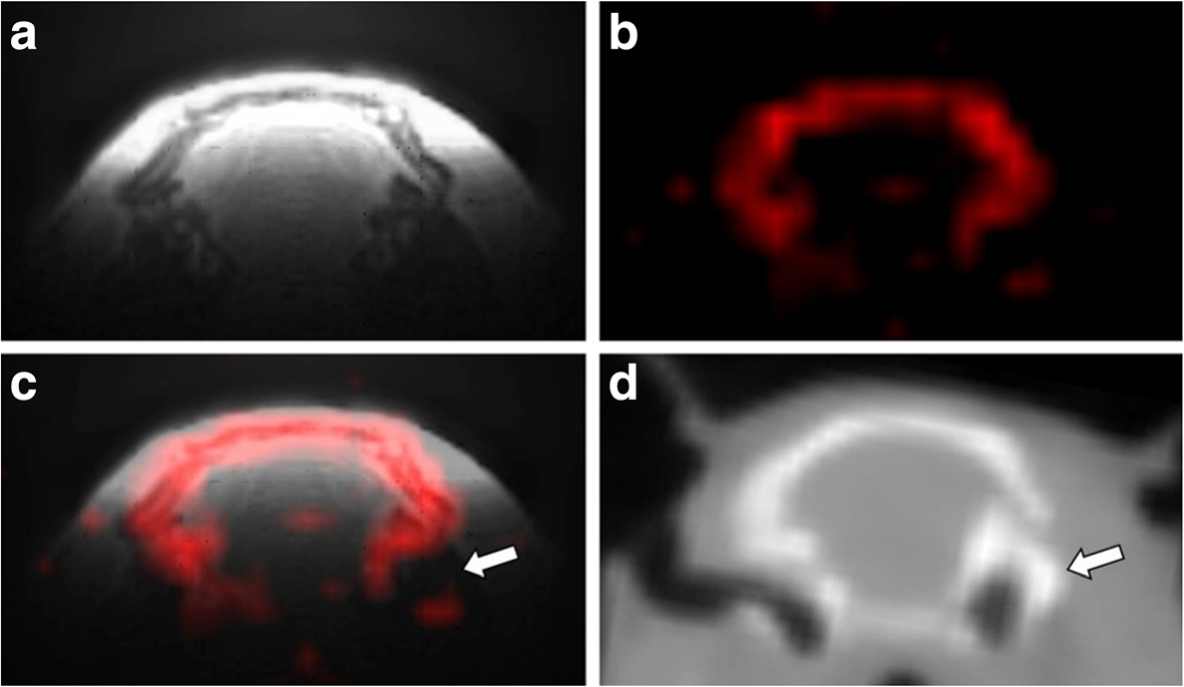
MRI and CT images acquired on the rat head in vivo. ^1^H anatomical MRI (**a**), ^31^P-ZTE MRI (**b**), fusion (**c**), and low resolution CT (**d**). The *white arrow* points to the skull base where ^31^P detection sensitivity is impaired

The MRI and CT data acquired in vivo are shown in Fig. 3, confirming good co-localization between the skull and ^31^P-ZTE signal and illustrating the bone specificity of ^31^P-ZTE MRI (no significant ^31^P signal from soft tissues). As pointed on Figs. 3c, d, ^31^P-ZTE seems to “miss” bone spots at the skull base (white arrow). Similarly, “red spots” can be seen outside the skull on Figs. 3b, c. Focal increase in ATP-PCr concentration or soft tissue calcification could theoretically explain these spots. However, the most likely explanation is the contribution of electronic noise, given the low SNR of ^31^P-ZTE images (~10). A similarity index was calculated between ^31^P-ZTE and CT skull images, confirming overall good co-localization (0.92 Dice index).

**Fig. 4:**
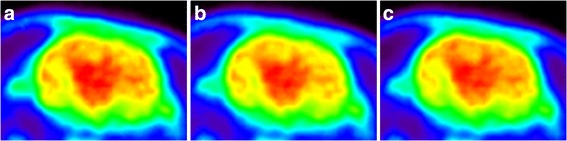
^18^F-FDG PET reconstructed using three different attenuation corrections: *CT-AC* (**a**), *NoBone-AC* (**b**), and ^*31*^ *P-AC* (**c**)

**Fig. 5:**
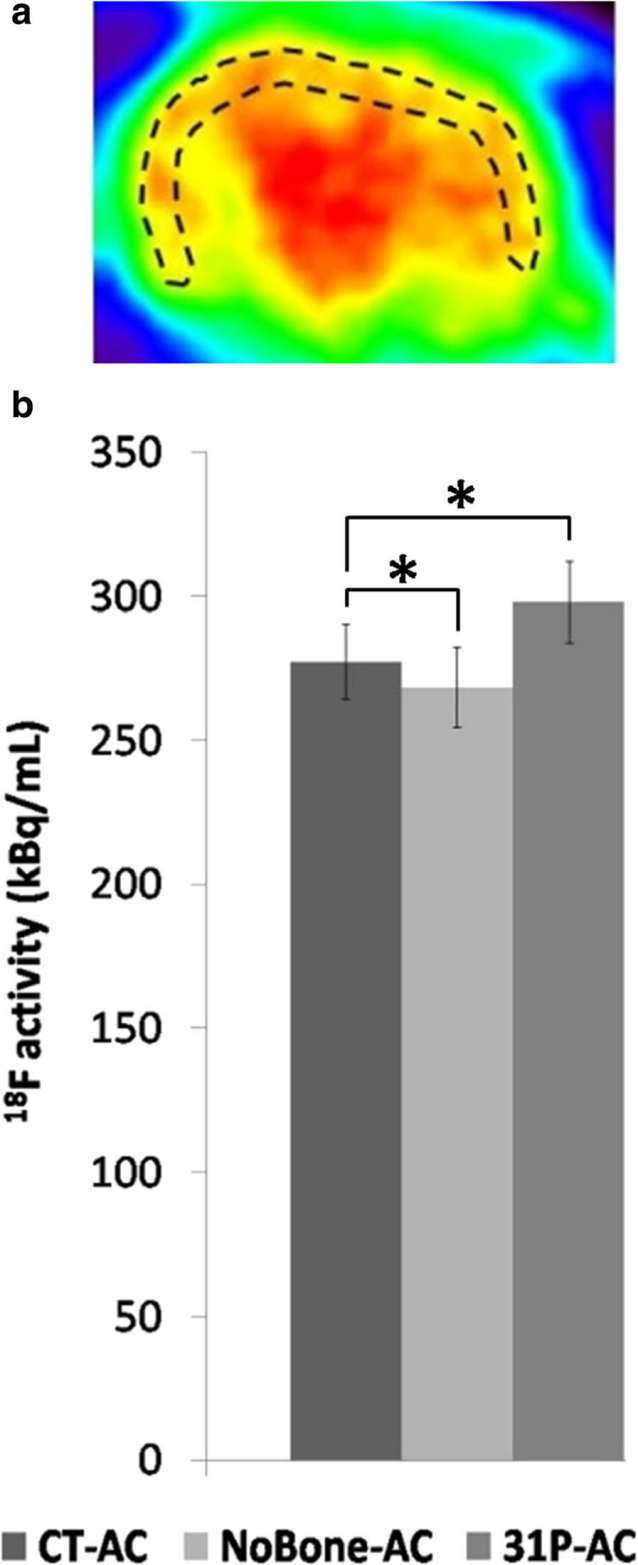
Peripheral brain ROI (**a**) in which ^18^F activity was measured for the three different attenuation corrections (**b**). *p < 0.001

Figure 4 displays ^18^F-FDG PET images of the rat brain reconstructed with the three different attenuation correction methods. In order to illustrate the effect of attenuation correction on brain PET images, ^18^F activity was measured in a peripheral brain ROI for the three sets of PET images (Fig. 5). As expected for such a small animal, skull attenuation had a limited impact on brain activity. However, Fig. 5 shows that NoBone-AC underestimates ^18^F activity as compared with *CT-AC* (*p < 0.001*, unpaired *t* test). The use of ^*31*^ *P-AC* significantly increases brain activity as compared with NoBone-AC and overestimates brain activity as compared with CT-AC (*p < 0.001*).

## Discussion

### Specificity of ^31^P-ZTE for the skull

As shown in Fig. 1, ^31^P is not only found in mineral hydroxyapatite in vivo but also in liquid-state molecules (mainly PCr and ATP). Moreover, the T1 of PCr or ATP is ~10 times smaller than the T1 of hydroxyapatite [17], such that the short TR used for ZTE is likely to enhance ^31^P signal of PCr and ATP. This raises the question of possible contamination of the signal of bone voxels by non-apatite molecules on ^31^P-ZTE images. Both theoretical and experimental data argue against such a contamination. Based on the molar mass of hydroxyapatite (402 g/mol for three phosphorus atoms) and on hydroxyapatite density in bone (3800 g/L), the concentration of ^31^P atoms in mineral bone can be assessed to ~30 mol/L. This is several orders of magnitude higher than PCr or ATP concentrations in soft tissues (~30 mmol/L for PCr in skeletal muscle [18]). Moreover, cortical bone is mainly made of hydroxyapatite crystals and collagen, with a low cell content [6] so that ATP and PCr concentrations in cortical bone are expected to be much smaller than in soft tissue. Therefore, the contribution of liquid-state molecules to the ^31^P signal of bone voxels is likely to be negligible as compared to the contribution of hydroxyapatite. This statement is supported by our experimental results: as illustrated by Figs. 3b, c, no significant ^31^P signal is detected in soft tissues on ZTE images of the rat head. This holds true for skeletal muscle which has the highest PCr and ATP contents among soft tissues [18] and is detected with a higher sensitivity than the skull due to the proximity of the muscle to the surface RF coil. This observation confirms earlier ^31^P-MRI studies of the wrist bone in vivo [15], showing no detectable signal from skeletal muscle.

### Quantitative measurement of bone mineral density

Our data demonstrate the ability to acquire in vivo images of skull hydroxyapatite using ^31^P-ZTE MRI. They do not demonstrate that ^31^P signal is quantitatively related to bone density. Such a demonstration would require the use of several bone samples with different densities and comparison with gold standard methods for bone density measurement, which was beyond the scope of this work. However, previous studies on bone samples have demonstrated that solid-state ^31^P-MRI is as good as or better than dual-energy X-ray absorptiometry in measuring bone mineral density by validation against chemical analysis [14]. Our approach relying on ^31^P-ZTE, it can therefore be considered to be quantitative for skull attenuation correction, as opposed to the currently available methods such as atlas-based, template-based, or segmentation-based methods [3].

### Homogeneity of MRI transmit and receive profiles

One limitation of our study is the use of surface coils for MRI acquisitions, resulting in a decreasing sensitivity from the cranial vertex to the skull base. This detection bias can be observed by comparing ^31^P-ZTE (Fig. 3c) and CT (Fig. 3d): part of the skull base seen on CT is missing on ^31^P-ZTE (white arrow). This bias is even more pronounced for the rat jaw which is not detected. However, the ^31^P coil is optimized for brain detection so that cortical bone is properly taken into account when located in close contact to the brain. It must be noted that the excitation profile may also vary with the flip angle. However, the transmission power was calibrated in order to maximize the broad peak of the ^31^P spectrum so that the effective flip angle was close to the Ernst angle of the bone, i.e., ~2°. At a low angle such as this one, the excitation profile is rather insensitive to the flip angle.

Another source of inhomogeneity of ^31^P signal is the frequency profile of the hard pulse used for excitation. A 15 μs hard pulse has a sinc-shape excitation profile for which the central lobe is ~130 kHz. Excitation can be considered to be reasonably homogeneous if all signals are located within the middle 35% of the profile (~45 kHz) where their intensity variation will be less than 5% of the maximum intensity. In practice, it means that 45/250 of the readout FOV will be properly excited, i.e., 16 mm. This value was adapted to our sample experiment (~8 mm largest dimension for the bone sample). For the in vivo experiment, the largest dimension of the skull is ~25 mm, for which the excitation profile cannot be considered to be homogeneous, leading to a signal decrease of 10–15% even when the skull is properly centered in the gradient coil.

### Attenuation correction of ^18^F-FDG PET images of the brain

Measurement of the ^18^F activity on reconstructed PET images shows an underestimation of brain activity when bone is neglected in the attenuation correction (Fig. 5). This observation is consistent with human studies reporting decreased cortical activity when the skull is assimilated with the soft tissue [4]. The use of ^31^P-ZTE for skull attenuation increases ^18^F activity as compared to the activity when NoBone-AC is used but also as compared to the activity when CT-AC is used. This reveals an overestimation of ^18^F activity by the ^31^P-based approach, corresponding to an overestimation of skull density. Besides measurement noise, a possible explanation for this overestimation is the inaccuracy of the conversion factor used to derive μ_31P_ from ^31^P-ZTE MRI: this factor was determined during a separate experiment performed on a bone sample imaged using the same MRI and CT systems. Different coil loading by the bone sample and the rat head might explain ^31^P sensitivity differences between the two experiments. Another explanation for inaccurate conversion factor could be chemical differences between the bovine sample and the rat skull. The attenuation of γ-photons by the bone is mostly due to calcium (atomic number *Z* = 20 versus *Z* = 15 for phosphorus): indeed interactions between photons and mineral bone are dominated by photoelectric effect for which μ∝*Z* ^4^ [19] so that attenuation by one Ca atom is ~3 times as high as attenuation by one P atom. Since ^31^P-ZTE detects phosphorus atoms, the conversion factor determined on our bovine sample would not apply to the rat skull if the Ca/P ratio were significantly different between species or bones. A literature review on the chemical properties of mineral bone reveals that hydroxyapatite Ca_5_(PO_4_)_3_OH is partly affected by lattice substitution, i.e., substitution of Ca^2+^ or PO_4_^3−^ by carbonate (CO_3_^2−^) or hydrogen phosphate (HPO_4_^2−^) [20]. However, lattice substitution affects only 5–10% of bone apatite [21]. In addition, a large majority of lattice substitution corresponds to carbonate located in PO_4_^3−^ sites [22, 23], with negligible impact on photon attenuation. Finally, the fraction of PO_4_^3−^ substitution by carbonate appears remarkably constant among vertebrate species [23]. Altogether, these observations argue in favor of a constant Ca/P ratio in cortical bone, close to the 5/3 stoechiometric ratio of hydroxyapatite.

### Limitations in rodent

This work has several technical limitations mostly due to the small size of the rat brain (~25 mm) in which MRI and PET detection sensitivities are significantly lower than in humans and attenuation of γ-photons is also much smaller than in humans. Moreover, the use of surface RF coils leads to significant bias in ^31^P sensitivity throughout the skull. Given these limitations, this work cannot claim to quantify the performance of ^31^P-based attenuation correction. In particular, difference maps or correlation scores—which are standard tools to compare attenuation corrections in humans [3, 24]—are dominated by biases and measurement noise. Indeed, none of the currently available methods for skull AC have been quantitatively assessed in small animals. This study presents an original approach, demonstrating the ability to obtain 3D images of the skull using ^31^P-ZTE MRI in vivo and presents an implementation for attenuation correction of brain ^18^F-FDG PET.

## Conclusions

This work brings theoretical and experimental arguments justifying the implementation and assessment of ^31^P-ZTE-based attenuation correction on human PET-MRI systems. The ^31^P approach should prove more straightforward and robust on a clinical imaging system. The first advantage of a human system will be the suppression of ^31^P-ZTE sensitivity in homogeneities. MRI of the human brain is performed with homogeneous volume RF coils, which are rarely used in rodent for geometrical reasons. Another advantage will be the suppression of the separate calibration experiment on bone sample, which is a major source of inaccuracy. The geometry of human RF coils will make it possible to insert a bone sample of known μ inside the ^31^P coil so that ^31^P signal can be directly converted into μ_31P_ by comparing the signal of the skull with the signal of the sample in the same image. Finally, implementation on a hybrid PET-MRI system will improve the correction accuracy over our experimental setup by suppressing bias associated with registration errors. Implementation on a human hybrid PET-MRI system will make it possible to carefully compare the ^31^P-based method with currently available methods.

## Abbreviations

CT: Computed tomography
FDG: Fluorodeoxyglucose
MRI: Magnetic resonance imaging
NMR: Nuclear magnetic resonance
PET: Positron emission tomography
RF: Radiofrequency
ZTE: Zero echo time
